# Biophysical Characterization of Met-G-CSF: Effects of Different Site-Specific Mono-Pegylations on Protein Stability and Aggregation

**DOI:** 10.1371/journal.pone.0042511

**Published:** 2012-08-08

**Authors:** Antonino Natalello, Diletta Ami, Maddalena Collini, Laura D’Alfonso, Giuseppe Chirico, Giancarlo Tonon, Silvia Scaramuzza, Rodolfo Schrepfer, Silvia Maria Doglia

**Affiliations:** 1 Department of Biotechnology and Biosciences, University of Milano-Bicocca, Milan, Italy; 2 Consorzio Nazionale Interuniversitario per le Scienze Fisiche della Materia (CNISM), UdR Milano-Bicocca, Milan, Italy; 3 Department of Physics, University of Milano-Bicocca, Milan, Italy; 4 IRCSS Multimedica, Milan, Italy; 5 Bio-Ker srl c/o Sardinia Scientific and Technological Park, Pula, Italy; Cardiff University, United Kingdom

## Abstract

The limited stability of proteins in vitro and in vivo reduces their conversion into effective biopharmaceuticals. To overcome this problem several strategies can be exploited, as the conjugation of the protein of interest with polyethylene glycol, in most cases, improves its stability and pharmacokinetics. In this work, we report a biophysical characterization of the non-pegylated and of two different site-specific mono-pegylated forms of recombinant human methionyl-granulocyte colony stimulating factor (Met-G-CSF), a protein used in chemotherapy and bone marrow transplantation. In particular, we found that the two mono-pegylations of Met-G-CSF at the N-terminal methionine and at glutamine 135 increase the protein thermal stability, reduce the aggregation propensity, preventing also protein precipitation, as revealed by circular dichroism (CD), Fourier transform infrared (FTIR), intrinsic fluorescence spectroscopies and dynamic light scattering (DLS). Interestingly, the two pegylation strategies were found to drastically reduce the polydispersity of Met-G-CSF, when incubated under conditions favouring protein aggregation, as indicated by DLS measurements. Our in vitro results are in agreement with preclinical studies, underlining that preliminary biophysical analyses, performed in the early stages of the development of new biopharmaceutical variants, might offer a useful tool for the identification of protein variants with improved therapeutic values.

## Introduction

The advent of recombinant DNA technologies allowed the production of a large number of proteins with potential applications as therapeutic drugs. However, several shortcomings might limit their usefulness as biopharmaceuticals. First of all, proteins have to be stable under storage conditions in order to avoid the formation of protein aggregates that could be associated to higher immunogenicity. Furthermore, proteins can be degraded in vivo by cellular proteases and/or rapidly excreted by kidneys, leading to a short circulating half-life that reduces their therapeutic efficacy [1]. To overcome these problems several strategies have been explored, as the use of protectants to increase the protein storage stability, the site-specific mutations to reduce the aggregation propensity and enzymatic proteolysis, and the incorporation of the protein within delivery vehicles. Moreover, the conjugation with polyethylene glycol (PEG) could increase protein stability, improve the pharmacokinetic properties and reduce the immunogenicity of the therapeutic proteins. Indeed, PEG is recognized as a non-toxic and non-immunogenic polymer, approved by regulatory authorities for modification of biopharmaceuticals. It should be, however, noted that the final effects of pegylation will depend on the polymer characteristics (size and shape), the conjugation chemistry and the pegylation site, which, for instance, should be distant from the binding site of the protein with its target.

Contrary to the first generation of random pegylation, not always able to guarantee protein homogeneity, stability and activity, the second generation is mostly a site-directed pegylation, which enables the production of conjugated proteins with better defined characteristics. The most employed pegylation strategies for site-specific conjugation involve the addition of PEG to the N-terminal residue of the protein chain, or to a free cysteine, naturally present or genetically introduced. Moreover, in the case of site-directed enzymatic pegylation, it is possible to increase the choice of the pegylation target, making it possible to conjugate the polymer to the suitable protein site [2], [3].

Interestingly, site-specific pegylation obtained following different approaches could in principle lead to isomeric products with different characteristics, such as in the case of the human granulocyte colony stimulating factor (G-CSF), whose aggregation propensity has been found to be differently affected when pegylation was performed following different strategies [4]–[7].

In this work, we report a biophysical characterization of the non-pegylated and of two different site-specific pegylated forms of recombinant human methionyl-G-CSF, also known as Filgrastim, a protein of 18,799 Dalton belonging to the family of hematopoietic cytokines. Filgrastim is widely used as therapeutic agent in chemotherapy and bone marrow transplantation, since it regulates the production and the differentiation of hematopoietic progenitor cells [8], [9]. Considering, therefore, the relevant clinical applications of Filgrastim, a great effort has been devoted to the development of protein modifications that could improve both protein stability in vitro and its pharmacokinetic properties.

We performed our studies on the site-specific mono-pegylations of Met-G-CSF at the N-terminal methionine (Met-G-CSF-Met1-PEG) and at glutamine 135 (Met-G-CSF-Gln135-PEG), in order to compare products with a significant difference in the pegylation site.

Our investigations show that both the site-specific mono-pegylations of Met-G-CSF preserve the protein secondary structure and strongly increase its thermal stability, as indicated by circular dichroism (CD), Fourier transform infrared (FTIR) and intrinsic fluorescence spectroscopies. Moreover, we found that both kinds of pegylation reduce the Filgrastim aggregation propensity and prevent its precipitation, as revealed by dynamic light scattering (DLS), CD and FTIR spectroscopies. In particular, by means of DLS we found that the two pegylation strategies drastically reduced the polydispersity of Met-G-CSF, when incubated under conditions favouring protein aggregation.

## Results

### Preparation and Characterization of Test Products

The products Met-G-CSF and Met-G-CSF-Met1-PEG, respectively corresponding to Filgrastim and to the commercial N-terminally mono-pegylated Filgrastim, were prepared according to the methods described in the literature [10] and in (Kinstler OB, Gabriel N, Farrar CE, De Prince RB (1998) N-terminally chemically modified protein compositions and methods. U.S. Patent 5,824,784), while Met-G-CSF-Gln135-PEG, an isomeric form of mono-pegylated Filgrastim, was prepared by site-specific enzymatic pegylation catalysed by a microbial transglutaminase. The selectivity of enzymatic pegylation is demonstrated by the fact that Met-G-CSF-Gln135-PEG, obtained with more than 90% yields, is mono-pegylated specifically only on the glutamine residue 135 over the seventeen glutamine residues of Met-G-CSF (Scaramuzza S, Olianas A, Caliceti P, Schrepfer R, Tonon G, Orsini G, A new site-specific mono-pegylated Filgrastim derivative prepared by enzymatic conjugation. 1. Production and chemico-physical characterization. Manuscript in preparation).

**Figure 1:**
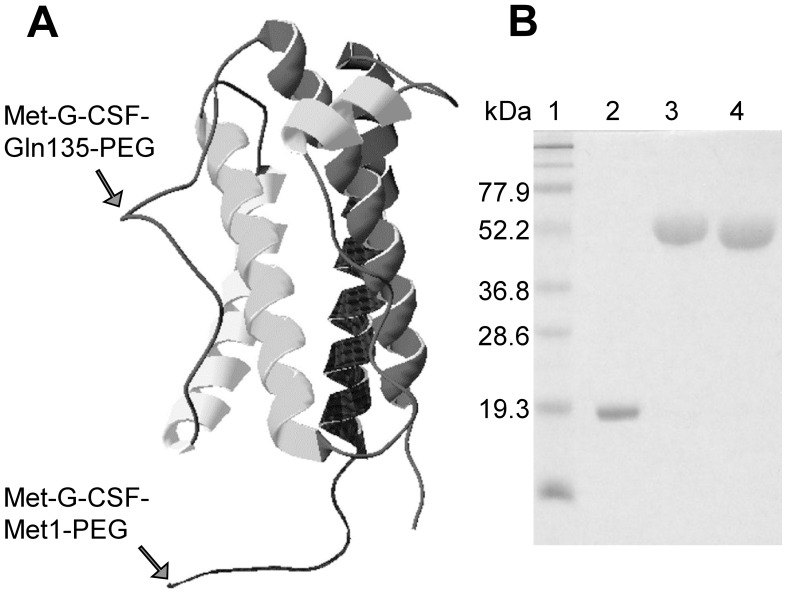
Structure of Met-G-CSF. A) Three-dimensional structure of Met-G-CSF (PDB ID: 1GNC) also showing (see arrows) the PEG conjugation sites of Met-G-CSF-Met1-PEG and Met-G-CSF-Gln135-PEG, which respectively are on N-terminal methionine and on glutamine 135 residue. B) Sodium dodecyl sulphate polyacrylamide gel electrophoresis of the non-pegylated Met-G-CSF and of the two pegylated variants, under reducing conditions. Lane 1, protein markers; lane 2, Met-G-CSF; lane 3, Met-G-CSF-Met1-PEG; lane 4, Met-G-CSF-Gln135-PEG.

The localization of the pegylation sites on the protein three-dimensional structure [11] is shown in Figure 1. The purity and homogeneity of the investigated proteins are also shown in Figure 1, where their SDS-PAGE separation is reported.

The biological activity of the products, measured by a proliferation assay, indicated that Met-G-CSF-Gln135-PEG had an in vitro residual proliferative activity of 17.6% (EC_50_ = 343.4±6.8 pg/ml), while Met-G-CSF-Met1-PEG of 13.8% (EC_50_ = 435.8±10.4 pg/ml), in comparison to non-pegylated Met-G-CSF, taken as 100% of activity (EC_50_ = 60.3±3.9 pg/ml).

As we will discuss later, we should note that protein pegylation very often leads to a decrease of the in vitro activity; however, this undesirable effect is generally offset by increased half-life and shelf-life [2].

### Met-G-CSF Secondary Structure by FTIR and CD Spectroscopies

**Figure 2:**
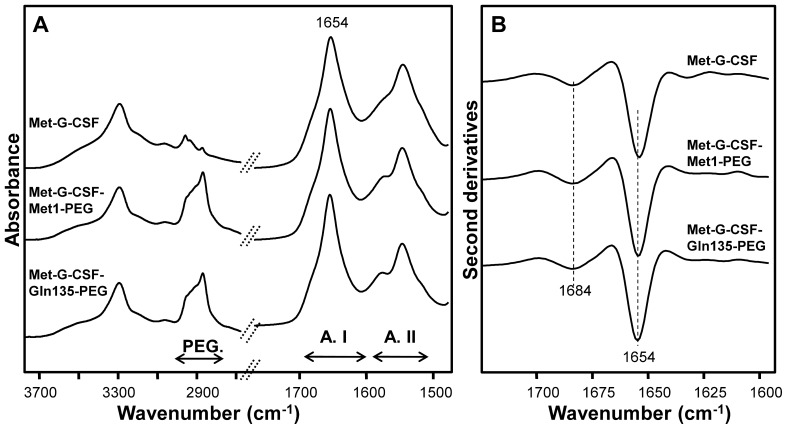
FTIR spectroscopy analysis of Met-G-CSF and of the two pegylated forms. A) FTIR spectra of non-pegylated Met-G-CSF and of the two isomeric Met-G-CSF-Met1-PEG and Met-G-CSF-Gln135-PEG. B) Second derivative spectra in the Amide I band of the three proteins as in A).

The non-pegylated Met-G-CSF and the two different site-specific pegylated forms were characterized by FTIR spectroscopy, a technique that allows to study the protein secondary structure and aggregation. In particular, the Amide I band between 1700 and 1600 cm^−1^, due to the CO stretching vibration of the peptide bond, is sensitive to the backbone conformational changes [12]–[14]. In Figure 2A the FTIR absorption spectra of the three tested compounds, measured at room temperature, are reported in the 3800–2700 cm^−1^ and 1900–1400 cm^−1^ spectral ranges. In particular, a higher absorption between 2950 and 2850 cm^−1^ in Met-G-CSF-Met1-PEG and Met-G-CSF-Gln135-PEG samples compared to the non-pegylated Met-G-CSF confirms the success of the pegylation reaction. Moreover, the three products display the same Amide I profile, indicating the preservation of the protein secondary structures, as better shown in Figure 2B where the second derivative spectra are reported. Indeed, this analysis [15] makes it possible to better resolve the protein secondary structure components, whose absorption overlaps in the broad Amide I band. The three samples are characterized by a dominant peak at 1654 cm^−1^ assigned to the protein α-helices, in agreement with the three-dimensional structure [11] of G-CSF (Figure 1) and with previous FTIR investigations [16] A second spectral component around 1684 cm^−1^ can be assigned to β-turn structures. Moreover, the absence of absorption between 1630–1620 cm^−1^, typical of intermolecular β-sheets, confirms that the proteins are in their native and non-aggregated form.

**Figure 3:**
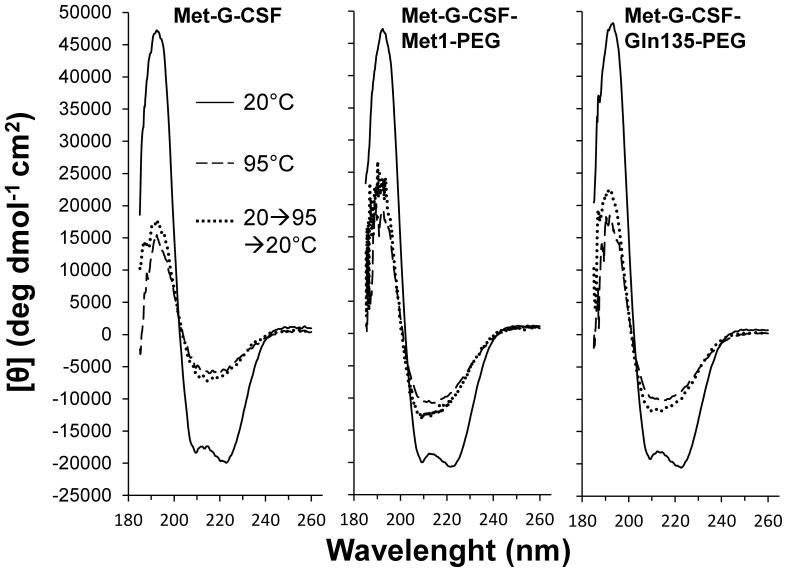
Thermal stability of Met-G-CSF and of the two pegylated forms by CD spectroscopy. CD spectra of non-pegylated Met-G-CSF and of the two isomeric Met-G-CSF-Met1-PEG and Met-G-CSF-Gln135-PEG at 20°C (continuous line), 95°C (dashed line), and 20°C after heating up to 95°C (dotted line).

These results were also confirmed by CD analysis [17] reported in Figure 3, where the three samples measured at 20°C display the same far UV spectra, characterized by two minima at ∼ 222 nm and ∼ 209 nm and a positive ellipticity at ∼ 193 nm, typical of α-helical proteins.

Overall, the FTIR and CD investigations indicate that the pegylation at both the protein N-terminal and at Gln135 doesn’t affect the protein native secondary structures.

### Thermal Stability by CD and Fluorescence Spectroscopies

We evaluated the thermal stability of non-pegylated and of the two different site-specific pegylated forms of Met-G-CSF by CD and fluorescence spectroscopies.

**Figure 4:**
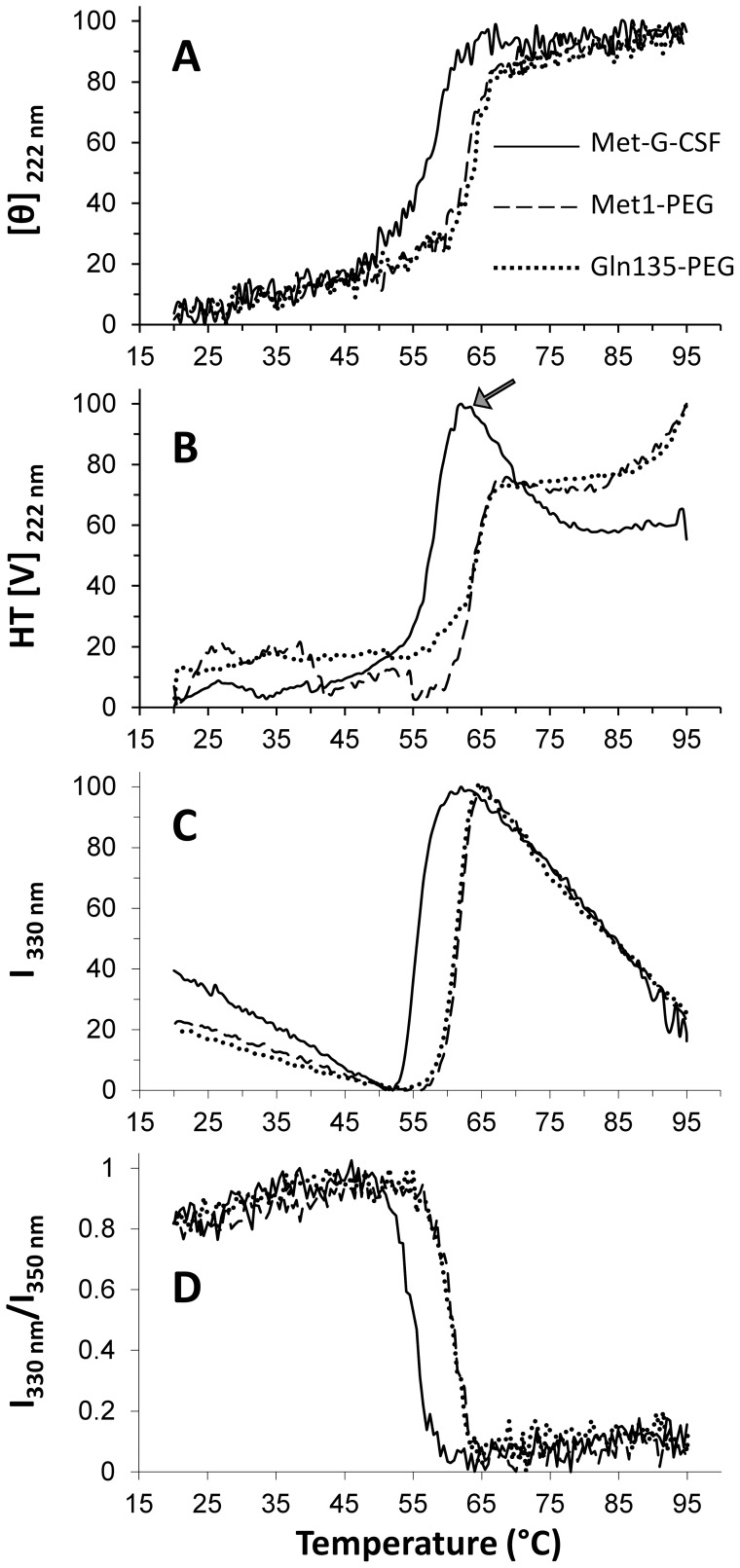
Thermal stability of Met-G-CSF and of the two pegylated forms by CD and fluorescence spectroscopies. A) Temperature dependence of the CD intensity at 222 nm. B) Temperature dependence of the HT[V] intensity at 222 nm. C) Temperature dependence of the fluorescence emission at 330 nm (excitation at 295 nm). D) Temperature dependence of the ratio between the fluorescence emission at 330 nm and at 350 nm (excitation at 295 nm).

In Figure 4A, the changes in the ellipticity at 222 nm are reported for the three samples, measured between 20°C and 95°C. In particular, the non-pegylated sample Met-G-CSF was found to be stable up to about 50°C, and then to rapidly lose its native structure with a transition mid-point at about 56°C. Interestingly, the two pegylated variants Met-G-CSF-Met1-PEG and Met-G-CSF-Gln135-PEG displayed a higher thermal stability, with a very similar transition mid-point around 62°C.

The direct inspection of the CD spectra at 95°C (Fig. 3) indicates that the three samples lost their native α-helical structure and acquired a CD signature typical of β-sheet structures, more evident in the non-pegylated form, characterized by a well resolved minimum at ∼ 217 nm [17]. This transition was also found to be irreversible, as shown by the CD spectra measured after cooling the samples to 20°C, displaying the same CD response as those obtained at 95°C (Fig. 3). During thermal unfolding, protein aggregation occurred, as indicated by the HT[V] changes (Fig. 4B), that allow to monitor the variation of the solution turbidity related to the formation of protein soluble aggregates [18]. In particular, the HT[V] plot at 222 nm (Fig. 4B) has been found to follow the same behavior of the ellipticity at the same wavelength (Fig. 4A), indicating that the thermal unfolding of the three Met-G-CSF variants is accompanied by protein aggregation. Noteworthy, while the non-pegylated Met-G-CSF was precipitating above 60°C (see the arrow in Fig. 4B), no protein precipitation was observed for the two pegylated derivatives, up to 95°C.

These results indicate that pegylation, independently of its localization site, not only increases the thermal stability of Met-G-CSF, but also avoids the formation of insoluble protein aggregates.

The thermal stability of non-pegylated Met-G-CSF and of the two different site-specific pegylated forms was also studied by fluorescence spectroscopy. In particular, we followed the fluorescence emission after excitation of the two Met-G-CSF tryptophans (Trp59 and Trp119) at 295 nm, during the thermal treatment. The emission intensity at 330 nm of the three Met-G-CSF variants, between 20°C and 95°C, is compared in Figure 4C. The non-pegylated Met-G-CSF sample displayed a linear reduction of the fluorescence intensity up to ∼ 50°C, likely due to quenching of the fluorescence as a consequence of increasing temperatures [19], [20]. Instead, at higher temperatures a rapid increase of the signal indicates the occurrence of protein structural rearrangements. Indeed, in the native protein the two Trp residues displayed a very low emission likely due to their interaction with charged amino-acids, as histidines, possibly responsible of their fluorescence quenching. Above ∼ 62°C the signal again linearly decreases, as typically occurs at increasing temperatures.

To better evaluate the protein mid-point of transition, we reported the ratio between the fluorescence emission intensity at 330 nm and at 350 nm [21]. In this way, the non-pegylated Met-G-CSF was found to be characterized by a transition mid-point of ∼ 56°C, in agreement with the CD results. We then performed the same analysis for the two pegylated variants that were found to be more stable than Met-G-CSF, both displaying a transition mid-point of ∼ 62°C, again in agreement with CD results.

### Protein Aggregation Detected by DLS and FTIR Measurements

**Figure 5:**
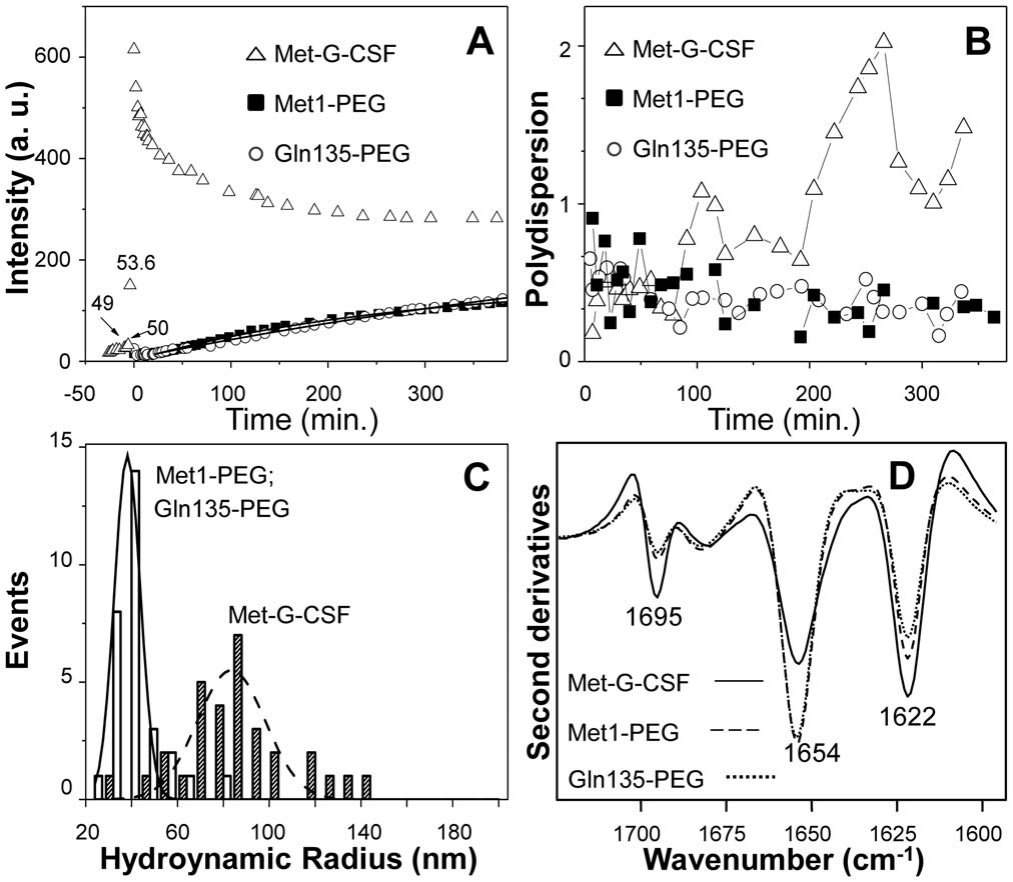
Dynamic light scattering and second derivative FTIR spectra of non-pegylated Met-G-CSF and of the two isomeric Met-G-CSF-Met1-PEG and Met-G-CSF-Gln135-PEG. A) Light scattering intensity, in arbitrary units, as a function of the incubation time at 55°C. At the zero time the bath temperature was changed from 37°C to 55°C. B) Polydispersity index (Eq.3) of the non-pegylated Met-G-CSF and of the two isomeric pegylated proteins as a function of the incubation time. C) Distribution of the hydrodynamic radii corresponding to the most abundant component in the DLS decay. The solid and dashed lines are best fit Gaussian function to the data that corresponds to Rh = 39.4±4 nm and Rh = 70±20 nm for the pegylated and non-pegylated samples respectively. D) Met-G-CSF, Met-G-CSF-Met1-PEG and Met-G-CSF-Gln135-PEG second derivative FTIR spectra measured after 7 hours of incubation at 55°C.Concerning the size of the protein aggregates, the diffusion coefficients (Eq.1) were used to evaluate the protein aggregate hydrodynamic radii through Eq.2. Non-pegylated Met-G-CSF displayed an average hydrodynamic radius of 1.4±0.4 nm at 37°C with negligible presence of protein aggregates. At 55°C, after thermal equilibrium was reached, we found instead a prevalent component with R_h_ = 70±20 nm. It should be noted that the value of 70 nm represents the average size of protein aggregates still in solution after 7 hours of incubation at 55^o^C.

We compared the aggregation kinetics of the non-pegylated Met-G-CSF and of the two pegylated variants Met-G-CSF-Met1-PEG and Met-G-CSF-Gln135-PEG to better understand the protection role of the pegylation against aggregation, in particular at the N-terminal and at Gln135. To this aim, the aggregation kinetics of the proteins were followed by DLS, starting at a temperature of 37°C and then rapidly (10 min) heating the samples at 55°C, close to the transition mid-point of the Met-G-CSF non-pegylated form. We monitored the protein aggregation kinetics at 55°C for about 7 hours, following the scattering intensity of the three samples as a function of time. As reported in Figure 5A, we found that the non-pegylated Met-G-CSF was characterized by an abrupt increase of the scattering intensity between 50°C and 55°C, which started to decrease right after (2 min) incubation at this temperature, reaching a plateau in about 2.5 hours. These results indicate that the non-pegylated protein, brought to a temperature of 50°C–55°C, formed highly scattering aggregates. The ≅ 20 fold increase in the scattering intensity observed in Figure 5A (open triangles), when rising the temperature above 50^o^C, suggests that large aggregates were forming upon bringing Met-G-CSF at temperatures of the order of 50^o^C. It is likely that precipitation of the largest aggregates took place at 55^o^C, a process that causes the observed slow decrease of the average intensity at this temperature. Noteworthy, the two pegylated variants Met-G-CSF-Met1-PEG and Met-G-CSF-Gln135-PEG displayed almost an identical profile of their scattering intensity, that is characterized instead by a slow monotonous increase in intensity that reached a plateau value significantly lower than that of the non-pegylated variant (Fig. 5A).

The two pegylated proteins displayed a very similar behavior in terms of hydrodynamic radius that was found to be R_h_ = 3.4±0.4 nm at equilibrium at T = 37^o^C and to rise to R_h_ = 39.4±4 nm after 7 hours incubation at 55°C. It is noteworthy that the observed monotonous increase in the scattering intensity as a function of the incubation time (Fig. 5A) corresponded to an increase in the fraction of the aggregates (R_h_ = 39.4±4 nm) versus time, rather than to an increase in their size.

These observations were confirmed by the polydispersity analysis (Eq. 3 and Figs. 5B and 5C). The polydispersity index, σ, of the three samples gives information on the heterogeneity of the protein soluble assemblies [22], [23]. In particular, at the beginning of incubation at 55°C, the non-pegylated protein displayed relatively low values of the polydispersity index, that dramatically increased with incubation time at 55^o^C (Fig. 5B). We should note that the value obtained here for σ refers to the residual soluble protein, since precipitation of the non-pegylated variant occurred just after the incubation at 55°C, as discussed above (Fig. 5A). On the other hand, the two pegylated variants always displayed, during incubation at 55°C, a relatively low value of the polydispersity index that further decreased during the incubation (Fig. 5B), suggesting that the two pegylated samples reached a fairly well defined size of smaller aggregates. In Figure 5C, we reported the R_h_ distributions of the three Met-G-CSF variants at 55°C to better evaluate the differences in their polydispersity features.

To better characterize the structural properties of final aggregates, we studied by FTIR the three samples after 7 hours of incubation at 55°C (Fig. 5D). The second derivative spectra of the non-pegylated Met-G-CSF and of the two pegylated Met-G-CSF-Met1-PEG and Met-G-CSF-Gln135-PEG displayed a component at 1654 cm^−1^ assigned to residual α-helical structures, and two peaks at 1622 cm^−1^ and 1695 cm^−1^, both due to the formation of intermolecular β-sheets, typical of protein aggregates [24]–[27]. In particular, Met-G-CSF showed a stronger reduction of the native α-helices, accompanied by a higher level of aggregation, compared to Met-G-CSF-Met1-PEG and Met-G-CSF-Gln135-PEG that, instead, retained a higher α-helical content.

Overall these results demonstrate that both the mono-pegylations of Met-G-CSF, on the N-terminal methionine and on glutamine 135, similarly and successfully protect the protein against unfolding and precipitation, limiting their aggregation.

## Discussion

Methionyl-granulocyte-colony stimulating factor (Met-G-CSF) is a therapeutic recombinant protein approved under the brand name of Neupogen® for the treatment of febrile neutropenia often associated to cancer chemotherapy [9]. However, Neupogen as well as the Bio-Ker product BK0023, which is a potential Neupogen biosimilar, requires daily subcutaneous injection since it is rapidly cleared from blood circulation, due to a combination of renal filtration and receptor mediate endocytosis [28]. The limits of repetitive injections have been overcome by the development of a long-lasting Met-G-CSF derivative chemically conjugated on N-terminal methionine residue with a 20 kDa PEG chain, whose clinical use, under the brand name of Neulasta®, was authorized by USA and European regulatory authorities in 2002. Clinical studies have in fact demonstrated that a single subcutaneous injection of Neulasta®, administered once per chemoterapy cycle, is no less effective than the daily administration of Filgrastim [29], indicating an increased half-life of the pegylated variant.

Bio-Ker has produced an isomeric form of Met-G-CSF-Met1-PEG by a site-specific enzymatic pegylation of Filgrastim, catalysed by a bacterial transglutaminase, to obtain the glutamine 135 pegylated Met-G-CSF (Tonon G, Orsini G, Schrepfer R, Taylor G, Sergi M (2009) G-CSF site-specific monoconjugates. EU Patent EP2049566 B1) (Fig. 1). Interestingly, the two pegylated forms were found to share comparable in vitro biological activity.

In this work, we characterized by several complementary biophysical approaches the non-pegylated and the two pegylated variants of Met-G-CSF, with the aim to disclose the effects of these modifications on the structural properties, stability and aggregation of the proteins.

First of all, we assessed, by FTIR and CD, that the two pegylation strategies didn’t affect the protein secondary structure. This point is, indeed, crucial since the protein function is known to be strictly related to the preservation of its native structure.

Concerning the protein stability, the thermal unfolding experiments performed by CD and fluorescence spectroscopies showed that both kinds of pegylation increase the stability of Met-G-CSF in the same way for the two pegylated products. This result is of great relevance since - as reported in the literature - pegylation could lead to products with different stability depending on the pegylation strategies [4]–[7].

In our work, we found that the thermal unfolding of the Met-G-CSF variants was associated to protein aggregation, as revealed by DLS, CD and FTIR spectroscopies. In particular, the non-pegylated protein underwent aggregation immediately after the temperature increase to 55°C, forming insoluble aggregates. Instead, the two pegylated proteins were characterized by a lower aggregation kinetics, that also led to soluble aggregates. Moreover, as studied by DLS, the aggregation at 55°C of the two pegylated products was found not to be associated to the formation of assemblies of increasing size, but to that of homogeneous size particles, with hydrodynamic radius of about 40 nm. Indeed, opposite to what found for the unmodified protein, both kinds of pegylation significantly reduced the polydispersity of the protein even at 55°C, up to the end of our observations (Figs. 5B and 5C).

These results, overall, indicate that the coupling of polyethylene glycol to Met-G-CSF allowed to obtain a considerable reduction in protein aggregation compared to the non-pegylated protein, both in terms of amount and size of aggregates, and in particular without the formation of insoluble aggregates. These properties enable to prepare a more concentrated pharmaceutical product with an improved shelf-life.

The different behavior of the two pegylated variants compared to the unmodified protein could be explained considering the steric hindrance of the PEG moiety, that reduces the aggregation rate, while the solvation by water molecules of the polar PEG groups increases the solubility of the pegylated protein assemblies.

### Conclusions

The possibility to control protein aggregation represents an important challenge in the development of protein for therapeutic use. Indeed, aggregation, that can occur during different stages of recombinant protein productions - from the expression in the host cells to the storage conditions - strongly affects protein bioavailability, immunogenicity and pharmacokinetics [1].

The results reported in this work show that both the site-specific pegylation strategies employed in this study led to two isomeric mono-pegylated Met-G-CSF derivatives with comparable structural properties and improved stability, associated to a lower aggregation propensity compared to the non-pegylated protein. Interestingly, these results are in agreement with preclinical studies (Crobu D, Schrepfer R, Tonon G, Orsini G, Preclinical pharmacokinetics and pharmacodynamics of a new site-specific mono-pegylated Filgrastim derivative prepared by enzymatic conjugation. Manuscript in preparation), that demonstrated for the two pegylated products superimposable pharmacodynamic and toxicological characteristics.

In this view, the biophysical approach described here might enable to evaluate protein stability and aggregation propensity of modified proteins, in the early stages of the development, offering a useful tool to identify samples with potential high therapeutic values.

## Materials and Methods

### Preparation of Test Products

#### Methionyl-G-CSF

Methionyl-G-CSF (INN Filgrastim, Bio-Ker company code BK0023) was produced by fermentation of a recombinant *E. coli* strain exploiting a fusion protein technology, as previously reported [10]. Briefly, the fusion protein LacZ_8_PNP_20_-Glu-Ser-Ser-Gly-Leu-Phe-Lys-Arg-Met-GCSF was expressed by high biomass fermentation as insoluble inclusion bodies that, after cell breakage, were recovered by centrifugation. After isolation of inclusion bodies, the fusion protein was dissolved in 7M guanidine buffer, refolded by dilution, dialysed and treated with the patented recombinant endoprotease ss-Kex1-C_611_ (Vanoni M, Tortora P, Tonon G, Taylor G, Orsini G (2006) Novel soluble endoproteases for the in vitro processing of recombinant proteins. EU Patent EP1334183 B1), which cleaved exactly at the carboxyterminal side of dipeptide Lys-Arg. The released Met-G-CSF was purified to homogeneity by three orthogonal column chromatographies and concentrated by ultrafiltration to about 3 mg/ml of Met-G-CSF.

#### N-terminal pegylated Met-G-CSF

The N-terminal pegylated Met-G-CSF (Met-G-CSF-Met1-PEG, Bio-Ker company code BK0025), corresponding to the commercial PegFilgrastim Neulasta®, was prepared by reductive alkylation of Met-G-CSF, as previously reported (Kinstler OB, Gabriel N, Farrar CE, De Prince RB (1998) N-terminally chemically modified protein compositions and methods. U.S. Patent 5,824,784). Briefly, monomethoxy-PEG-propionaldehyde 20 kDa (Sunbio, Orinda, CA, USA) was added under stirring to a cooled solution of Met-G-CSF in 100 mM pH 5 phosphate buffer containing 20 mM sodium cianoborohydride, at a molar ratio protein:PEG reagent of 1∶5. The one-pot reaction includes the PEG binding preferentially to the α-amino group as a Schiff base, which is reduced to a secondary amine by sodium cianoborohydride.

After overnight incubation under stirring, excess reagents and by-products (traces of di- and tri-pegylated derivatives) were separated by cation-exchange chromatography on a CM Sepharose column eluted with a NaCl linear gradient (from 0 to 500 mM in 15 column volumes). The fraction pool containing the purified Met-G-CSF-Met1-PEG was concentrated by ultrafiltration to about 10 mg/ml (expressed as protein moiety).

#### Gln135 pegylated Met-G-CSF

Gln135 pegylated Met-G-CSF (Met-G-CSF-Gln135-PEG, Bio-Ker company code BK0026) was prepared by a specific enzymatic transamidation between 20 kDa monomethoxy-PEG-alkylamine (Sunbio, Orinda, CA, USA) and the side chain of Met-G-CSF glutamine 135, catalysed by a microbial transglutaminase, as previously described (Tonon G, Orsini G, Schrepfer R, Taylor G, Sergi M (2009) G-CSF site-specific monoconjugates. EU Patent EP2049566 B1). Briefly, to a 2 mg/ml Met-G-CSF solution in 20 mM pH 8.1 potassium dihydrogen phosphate buffer, also containing 20 mg/ml of 20 kDa monometoxy-PEG-alkylamine, was added a solution of Activa WM microbial transglutaminase (Ajinomoto, Hamburg, Germany) to a final enzymatic activity of 0.25 U/ml. The solution was kept under stirring for 5 hours in refrigerator, diluted in 20 mM, pH 5.0 sodium acetate buffer and purified by cation exchange chromatography on a Macrocap SP column eluted with 98 mM sodium acetate buffer pH 5.0. The fraction pool containing Gln135 mono-pegylated Met-G-CSF was dialysed against 10 mM acetic acid pH 4.5 buffer and concentrated by ultrafiltration to about 13 mg/ml (expressed as protein moiety).

### In vitro Biological Activity

Biological activity of non-pegylated and pegylated Met-G-CSF derivatives was measured in vitro according to a described method [30], employing the murine myeloblastic leukemia cell line M-NSF60 (ATCC CRL-1338), which increases its proliferation activity in the presence of G-CSF. Briefly, M-NFS60 cells were distributed into 96-well plates at a concentration of 100 cells/well in 200 µl of RPMI 1640 medium - 10% fetal calf serum containing increasing concentration of Met-G-CSF (0.01–5 ng/ml), Met-G-CSF-Met1-PEG or Met-G-CSF-Gln135-PEG (1–30 ng/ml, calculated as Met-G-CSF equivalent). The plates were incubated at 37°C in a 5% CO_2_ atmosphere. After 48 hours, 20 µl of tetrazolium salt WST-1 were added to the cells and incubation continued further for 4 hours under the same conditions. The absorbance of formazan derived from WST-1 cleavage by cellular mitochondrial dehydrogenases was measured using an ELISA microplate reader (microplate reader model 680, Bio-Rad Laboratories Inc. Hercules, CA, USA), equipped with a 420–480 filter, against a buffer background. The biological activity of each sample was calculated from proliferation curve and expressed as EC_50_ value (concentration that stimulates 50% of maximal growth).

### Fourier Transform Infrared Spectroscopy Measurements

For FTIR measurements in attenuated total reflection (ATR), 5 µl of Met-G-CSF, Met-G-CSF-Met1-PEG or Met-G-CSF-Gln135-PEG, diluted at 2.7 mg/ml in 10 mM sodium acetate buffer pH 4.5, were deposited on the diamond plate of the single reflection ATR device (Golden Gate, Specac, USA). Spectra were recorded after solvent evaporation to allow the formation of a hydrated protein film [25]. The Varian 670-IR spectrometer (Varian Australia Pty Ltd., Mulgrave VIC, AU), equipped with a nitrogen-cooled Mercury Cadmium Telluride detector and an air dryer purging system, was employed under the following conditions: 2 cm^−1^ spectral resolution, 25 kHz scan speed, 512 scan coadditions, and triangular apodization. The second derivatives of the measured spectra [15] were obtained by the Savitsky-Golay method (third grade polynomial, 5 smoothing points) after binomial smoothing (11 points) using the GRAMS/AI software (Galactic Industries Corporation, Salem, NH, USA).

### Circular Dichroism Measurements

CD spectra in the far-UV region (185–260 nm) were recorded on a Jasco J-815 spectropolarimeter (Jasco Corp. Tokyo, Japan). Non-pegylated and pegylated Met-G-CSF variants were measured at 0.1 mg/ml, in 10 mM sodium acetate buffer pH 4.5, in a 0.1 cm path length quartz cell at a scanning speed of 10 nm/min and by the averaging of 4 scans.

For thermal unfolding experiments, the samples were heated from 20 to 95°C at a rate of 1°C /min and the ellipticity and the high tension voltage (HT[V]) at 222 nm were monitored every 0.5°C. CD spectra in the full far UV range were then collected at 20°C, 95°C, and at 20°C after heating up to 95°C.

### Intrinsic Fluorescence Measurements

Intrinsic fluorescence measurements were performed with a Cary Eclipse Varian fluorimeter (Varian Australia Pty Ltd., Mulgrave VIC, AU), with an excitation wavelength of 295 nm (slit 5 nm). Non-pegylated and pegylated Met-G-CSF variants were measured at 0.1 mg/ml, in 10 mM sodium acetate buffer pH 4.5 and in a 1 cm path length quartz cell.

For thermal unfolding experiments, the samples were heated from 20 to 95°C at a rate of 1°C/min and the fluorescence emissions were monitored at 330, 340, and 350 nm (slit 5 nm).

### Dynamic Light Scattering Measurements

Dynamic light scattering measurements have been performed for non-pegylated and pegylated Met-G-CSF variants on a home-made spectrometer [31]. The laser source was an Argon ion laser (2025, Spectra Physics, Mountain View, CA, USA) tuned at 514 nm, used at the average power of 50 mW. A cylindrical quartz cell (Hellma GmbH & Co, Germany) was thermostated (Thermo Haake GmbH, Germany) between 37°C and 55°C with a precision of 0.1^o^C. The temperature was monitored by a thermocouple placed just below the scattering cell.

The normalized intensity autocorrelation functions (ACFs) were computed by an ISS FCS board (ISS Inc. Urbana, IL, USA) and they were fitted to a multi-exponential decay law according to the following relation [32]:


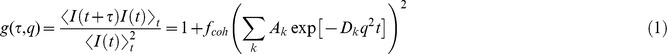


where D_k_ is the translational diffusion coefficient of the k-th diffusing species, q is the wave vector, q = (4πn)/(λ)sin(θ/2), n is the refraction index of the solution, λ = 514 nm is the laser light wavelength and θ = 90^o^ is the scattering angle. The parameter f_coh_ depends on the ratio between the detector and the coherence area, which was left as a free fitting parameter, typically in the range 0.35–0.15. The values of D_k_ provide us the average size of the diffusing objects, according to the Einstein relation:


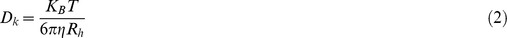


where KB is the Boltzmann constant, T and η are the solution temperature and viscosity and Rh is the hydrodynamic radius.

An alternative way to analyze the DLS data is to use the cumulant analysis. In order to gain information on the solution polydispersity [22], [23], we performed a fit of the data to a third order cumulant function:


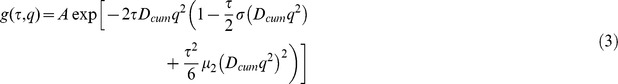


where σ is the polydispersity of the sample. It must be noted that the absolute value of the polydispersity index σ depends critically on the number of points used for the analysis of the autocorrelation function decay and the number of cumulants used to describe the decay. Here we have fitted Eq.3 to the autocorrelation function decay in the range 2–30 µs of lag times.
